# Direct and Indirect Determination of the Magnetocaloric Effect in the Heusler Compound Ni_1.7_Pt_0.3_MnGa

**DOI:** 10.3390/e23101273

**Published:** 2021-09-29

**Authors:** Ricardo D. dos Reis, Luana Caron, Sanjay Singh, Claudia Felser, Michael Nicklas

**Affiliations:** 1 Max Planck Institute for Chemical Physics of Solids, Nöthnitzer Str. 40, 01187 Dresden, Germany; luana.caron@uni-bielefeld.de (L.C.); ssingh.mst@iitbhu.ac.in (S.S.); Claudia.Felser@cpfs.mpg.de (C.F.); Michael.Nicklas@cpfs.mpg.de (M.N.); 2 Brazilian Synchrotron Light Laboratory (LNLS), Brazilian Center for Research in Energy and Materials (CNPEM), Campinas 13083-970, Brazil; 3 Faculty of Physics, Bielefeld University, P.O. Box 100131, 33501 Bielefeld, Germany; 4 Helmholtz-Zentrum Berlin für Materialien und Energie, Albert-Einstein-Straße 15, 12489 Berlin, Germany; 5 School of Materials Science and Technology, Indian Institute of Technology (BHU), Varanasi 221005, India

**Keywords:** first-order transitions, magnetocaloric effect, Heusler materials

## Abstract

Magnetic shape-memory materials are potential magnetic refrigerants, due the caloric properties of their magnetic-field-induced martensitic transformation. The first-order nature of the martensitic transition may be the origin of hysteresis effects that can hinder practical applications. Moreover, the presence of latent heat in these transitions requires direct methods to measure the entropy and to correctly analyze the magnetocaloric effect. Here, we investigated the magnetocaloric effect in the Heusler material Ni${}_{1.7}$Pt${}_{0.3}$MnGa by combining an indirect approach to determine the entropy change from isofield magnetization curves and direct heat-flow measurements using a Peltier calorimeter. Our results demonstrate that the magnetic entropy change ΔS in the vicinity of the first-order martensitic phase transition depends on the measuring method and is directly connected with the temperature and field history of the experimental processes.

## 1. Introduction

The Heusler compounds are known for their multiple phases, which make them promising materials for both technological applications and basic science. The connection between structural instabilities and magnetic degrees of freedom seems to be the key point for the multifunctional properties. Many exotic effects are observed in this class of materials, such as topological phases, giant magnetoresistance, extremely large exchange bias, non-collinear antiferromagnets, compensated ferrimagnets, semi-metallic ferromagnets, unconventional superconductors, anomalous Hall effect, caloric and shape-memory effects [1,2,3,4,5,6,7,8,9,10,11,12,13]. In particular, shape-memory materials have been widely studied in the context of magnetic refrigeration. On heating, these compounds undergo a first-order transition from a low-symmetry phase called ‘martensite’ (M) to a cubic phase called ‘austenite’ (A) at temperature $T_{M}$. This transition has a large latent heat and, consequently, a strong entropy increment. More recently, it is has been reported that, in materials with martensitic phase transitions, a precursor martensitic (PM) phase appears before the martensitic transition upon lowering temperature, starting from the austenite. Materials undergoing a (reverse) martensitic transition have attracted considerable attraction as potential magnetic refrigerants due to the large inverse magnetocaloric effect (MCE) associated with the magnetic-field-induced reverse martensitic transformation (martensite to austenite).

The stoichiometric compound Ni${}_{2}$MnGa has been intensively studied due to its large ≈10% magnetic field-induced strain (MFIS). The martensitic transition in Ni${}_{2}$MnGa is preceded by a precursor martensitic phase transition around $T_{pm}=260$ K. Both the martensite and paramagnetic phases exhibit very strong couplings between the two ferroic order parameters, i.e., magnetoelastic coupling, which is responsible for the large MFIS. The large MFIS is also closely linked with the existence of a long-period modulated structure of the martensite phase in Ni${}_{2}$MnGa. The A–PM and the PM–M transitions are first order in nature, with characteristic thermal hysteresis and phase coexistence regions, due to the strong magnetoelastic coupling between the magnetization and the strain caused by a soft TA2 phonon mode at a wave vector q≈1/3 [2,14].

The multiple phases and the flexibility of the Heusler structure makes this family of compounds ideally suited for optimizing the magnetic and structural phase transitions for magnetocaloric applications [15,16,17]. However, the practical application of giant MCE materials is necessarily hindered by the nature of the phase transition itself. Although the MCE is intrinsic to all magnetic materials, being particularly large in the vicinity of first- or second-order magnetic phase transitions, for the first-order transition, energy must be spent to overcome the potential barrier between the two phases. This leads to intrinsic irreversibilities in both isothermal entropy and adiabatic temperature changes, which can drastically reduce the cooling efficiency [18,19,20,21]. In order to optimize the cooling efficiency, huge efforts have been made to control the characteristics of the magnetic transition [22,23]. In particular, in NiMnGa, Pt-substitution on the Ni site appears as an excellent route, since one obtains a series of magnetic shape-memory alloys with properties similar to NiMnGa, but with a larger internal strain [2,11,14]. On the other hand, one of the open problems for the application of magnetic materials for magnetic cooling is the quantitative knowledge of the role played by hysteresis and irreversibility effects. In [24,25,26,27,28], since the entropy change calculated from magnetization might be overestimated when compared with the entropy change calculated from specific heat measurements. This is due to the different characteristics of each technique and measurement history before each isothermal magnetization measurement. To fully assess the MCE properties of materials with irreversible behavior, one can directly measure the heat flow, a physical quantity that is directly connected to the entropy change ΔS. Therefore, heat-flow methods emerge as an excellent alternative for the investigation of the MCE. In particular, those methods using small Peltier units to determine the heat flow have proven to be very effective [29,30,31,32]. In the present study, we will compare the entropy change ΔS in Ni${}_{1.7}$Pt${}_{0.3}$MnGa, obtained by the heat-flow method with ΔS calculated based on the Maxwell relation from isofield magnetization curves.

## 2. Materials And Methods

Polycrystalline ingots of Ni${}_{1.7}$Pt${}_{0.3}$MnGa were prepared by melting appropriate quantities of Ni, Pt, Mn, and Ga of 99.99% purity in an arc furnace. The ingots were then annealed at 1173 K for 3 days and subsequently quenched in ice water. The ingots were then annealed at 1173 K for 3 days and subsequently quenched in ice water. Homogeneity was confirmed by energy dispersive X-ray spectroscopy (EDX). The sample was found to be homogeneous with an average composition of Ni${}_{1.73}$Pt${}_{0.3}$Mn${}_{0.98}$Ga${}_{0.99}$. In the following, we use the nominal composition Ni${}_{1.7}$Pt${}_{0.3}$MnGa. The crystal structure at room temperature is found to be in the monoclinic-modulated martensite phase. More details on sample preparation and the structural properties of the sample can be found elsewhere [2,10,11].

The magnetic properties for fields up to 2 T have been obtained using a MPMS3 (Quantum Design). The isofield magnetization curves were measured at 3 K/min. The magnetothermal characterization was performed by a heat-flow method adapted to the PPMS (Quantum Design), as described elsewhere [30,33]. Here we used a standard bare puck for the PPMS, consisting of a metal base with an electrical connector underneath as a platform for the Peltier element. In our case, we used a $6\times 8\times 2.4{\;\mathrm{mm}}^{3}$ unit (Custom Thermoelectric, 032019G3008RA) with 32 thermocouple pairs. The calibration of the Peltier element followed the same procedure described in the literature [30,33]. To read the Peltier voltage we used the PPMS user bridge as a voltmeter. The heat-flow sensor voltage was recorded during both heating and cooling cycles with a temperature sweeping rate of 0.5 K/min between 190 and 350 K for the empty sample platform and for a high purity copper sample as a standard. With this procedure we determined the heat capacity $C_{sys}\left(T\right)$ of the measurement setup and the sensitivity of the Peltier element A(T) (with A=S/K, where *S* is the Seebeck coefficient and *K* is the thermal conductance of the Peltier element; $A\left(T\right)/\left[\mu V/K\right]=0.2792-0.0027\times T+1.68464\;\times 10^{-5}\times T^{2}-2.36472\;\times 10^{-8}\times T^{3}$). We discarded the initial data of the temperature sweeps (about 10 K) to guarantee a constant temperature sweeping rate of 0.5 K/min.

We used similar measurement protocols for both employed experimental probes, magnetization and heat flow. Initially, the sample was cooled to the base temperature well inside the martensite phase. Then, the data were recorded by heating the sample to the maximum temperature well in the austenite phase. Once the maximum temperature was reached, we waited 10 min for a complete thermalization of the system and started the cooling run down to the minimum temperature. After a period of 10 min for thermalization, a magnetic field was applied, and heating and cooling runs were carried out again, followed by the next field. For the heat-flow measurements, the experiments were performed by increasing the magnetic fields at 1 and 2 T. The magnetization runs were recorded upon decreasing the magnetic field in several steps from 2 T down to 0.1 T.

## 3. Results

**Figure 1:**
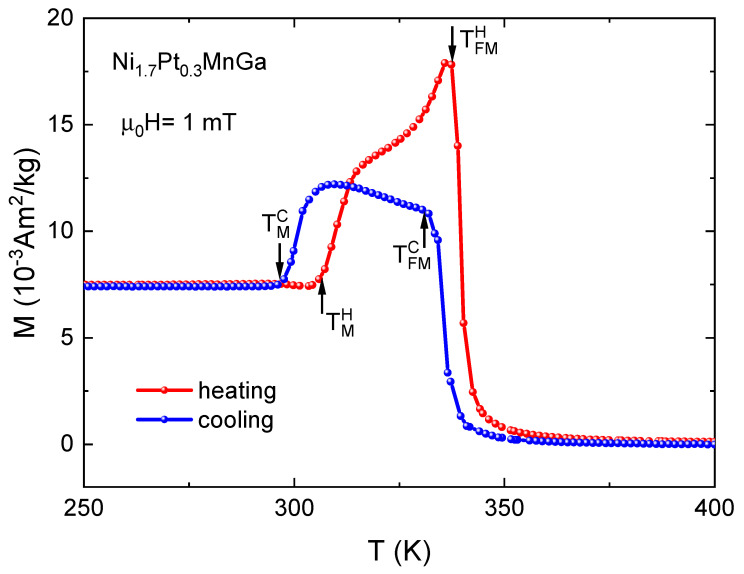
Low-field ($\mu_{0}H=1$ mT) magnetization curves for cooling and heating cycles for Ni${}_{1.7}$Pt${}_{0.3}$MnGa exhibiting structural and magnetic transitions.

The temperature-dependent magnetization curves M(T) of Ni${}_{1.7}$Pt${}_{0.3}$ MnGa measured in both cooling (blue) and heating (red) cycles in a small magnetic field of 1 mT are shown in Figure 1. We obtained the martensitic and ferromagnetic transition temperatures upon cooling (heating) $T_{M}^{C(H)}$ and $T_{FM}^{C(H)}$ from the M(T) curves. Upon cooling, the paramagnetic–ferromagnetic transition started at 342 K, followed by a sharp increase in the magnetization until 331 K. After that, M(T) still increased, but with a strongly reduced rate until we reached the temperature where a sudden drop in the magnetization indicates the martensitic transition. The martensitic finish temperature $T_{M}^{C}=296$ K. The peak and drop in the M(T) curve are caused by the large magnetocrystalline anisotropy of the martensite phase. We observed similar features in M(T) for the heating cycle; however, this was with a pronounced hysteretic behavior between both transitions. For the ferromagnetic–paramagnetic transition, the hysteresis width was about 4 K; for the martensitic transition, the hysteresis width was significantly larger, around 10 K. The hysteresis was taken as the difference between the respective starting temperatures on heating and the finish temperatures on cooling (see Figure 1). The hysteresis width observed in the present work was larger in comparison with previously reported results [2]. We note that we recorded M(T) at a very small magnetic field of only 1 mT. As the magnetic field stabilizes the phase with the higher magnetic moment—in this case the martensitic phase—we expect the hysteresis of the inverse transition to increase with increasing field.

**Figure 2:**
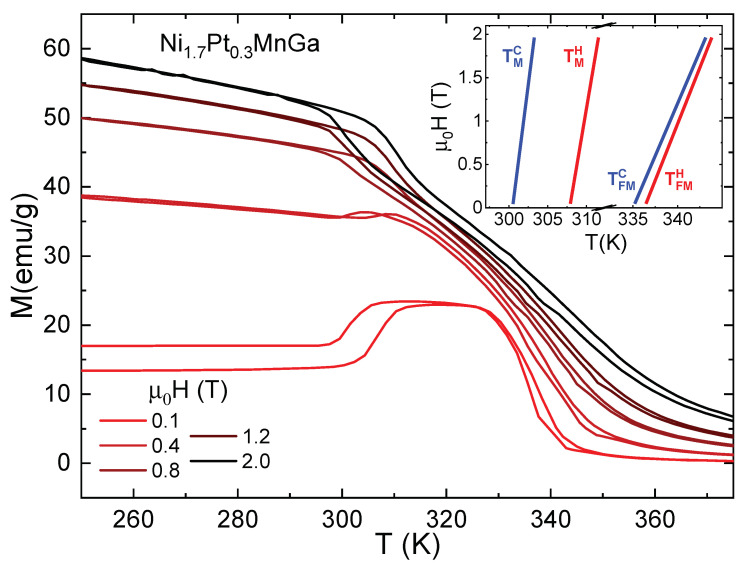
Selected isofield magnetization curves at different fields between 0<H≤2 T, as indicated, on both heating and cooling for Ni${}_{1.7}$Pt${}_{0.3}$MnGa exhibiting structural and magnetic transitions. The inset illustrates the H−T phase diagram.

Figure 2 shows the effect of the magnetic field on the magnetization M(T), measured upon heating and cooling in various magnetic fields up to 2 T in the vicinity of the martensitic phase transition. As expected, the external magnetic field shifts the martensitic transition towards lower temperatures, thus stabilizing the ferromagnetic austenitic phase.

Ni${}_{1.7}$Pt${}_{0.3}$MnGa shows a second-order and a first-order phase transition, with thermal hysteresis in both. In the following, we investigate both transitions in detail. First we present the magnetic entropy ΔS obtained from the isofield magnetization curves. According to classical thermodynamics, the magnetic entropy change ΔS as a consequence of an increase in magnetic field can be indirectly determined by using the magnetization curves displayed in Figure 2 and the following expression:

(1) $$\Delta S=S\left(T,H\right)-S\left(T,H=0\right)=\int_{0}^{H}{\left[\frac{\partial M(T,H^{\prime })}{\partial T}\right]}_{H^{\prime }}dH^{\prime }$$

Hereafter, ΔS is defined as the entropy change upon variation in the applied magnetic field at a constant temperature. It includes magnetic, electronic, and structural contributions. It is worth noting that the MCE in the vicinity of the martensitic transition of the Ni${}_{2}$MnGa family of compounds has, so far, been studied mostly for heating runs, i.e. for the case of the reverse martensitic transformation from the martensitic to the austenitic phase. However, the characteristic temperatures of the direct martensitic transformation are lower than those of the reverse one. This affects the magnitude of the magnetization change across the transitions. Moreover, the direct martensitic transformation is an exothermic process, whereas the reverse one is endothermic. Evidently, the magnetocaloric properties of a ferromagnetic shape-memory alloy upon direct and reverse transformations will not be the same.

**Figure 3:**
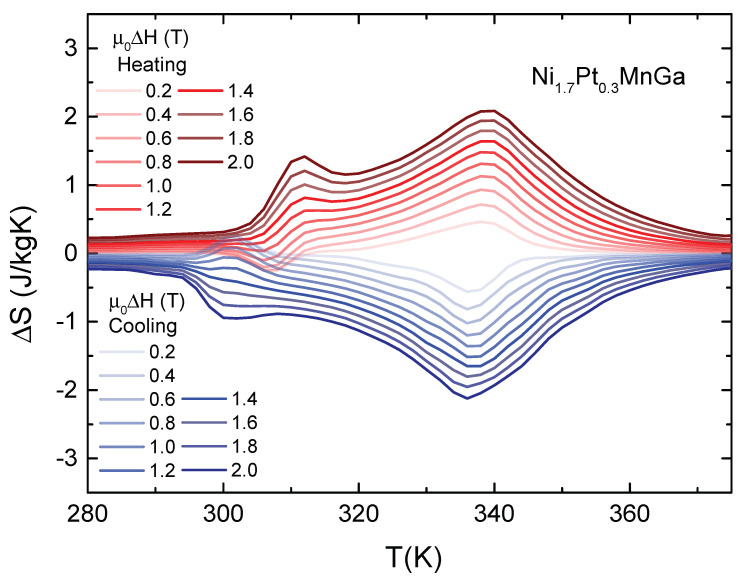
Magnetic entropy change ΔS as a function of temperature for different field changes $\mu_{0}\Delta H$ of Ni${}_{1.7}$Pt${}_{0.3}$MnGa.

The isothermal entropy changes ΔS for heating and cooling, calculated from M(T) for magnetic field changes from 0 to 0.2 T, from 0 to 0.4 T and so forth up to 2 T in 0.2 T steps, are depicted in Figure 3. ΔS exhibits two peaks centered at 310 K and 340 K, which are related to the martensitic $\Delta S_{M}$ and the paramagnetic–ferromagnetic $\Delta S_{FM}$ transitions, respectively. For a magnetic-field change of 0.2 T, we find, upon heating, $\Delta S_{M}=-0.2$ J/kgK and $\Delta S_{FM}=0.4$ J/kgK. The peak at the ferromagnetic–paramagnetic transition increases continuously upon increasing the magnetic field change, reaching $\Delta S_{FM}=2.1$ J/kgK for $\mu_{0}\Delta H=2$ T, while $\Delta S_{M}$ changes sign reaching $\Delta S_{M}=1.5$ J/kgK for $\mu_{0}\Delta H=2$ T. Moreover, we found that, although there is a slight shift in the temperature position of the peaks, the amplitude of ΔS is quite similar for both heating and cooling cycles.

In order to further investigate the role played by the first-order nature of the martensitic transition and the effect of the magnetic transitions on the properties of the MCE in Ni${}_{1.7}$Pt${}_{0.3}$MnGa, we performed direct heat-flow measurements using a Peltier calorimeter. The basic idea of this method is to use a Peltier element as a heat-flow sensor (instead of a heat pump), which allows for the determination of the total heat exchange between the sample and the heat reservoir [33].

**Figure 4:**
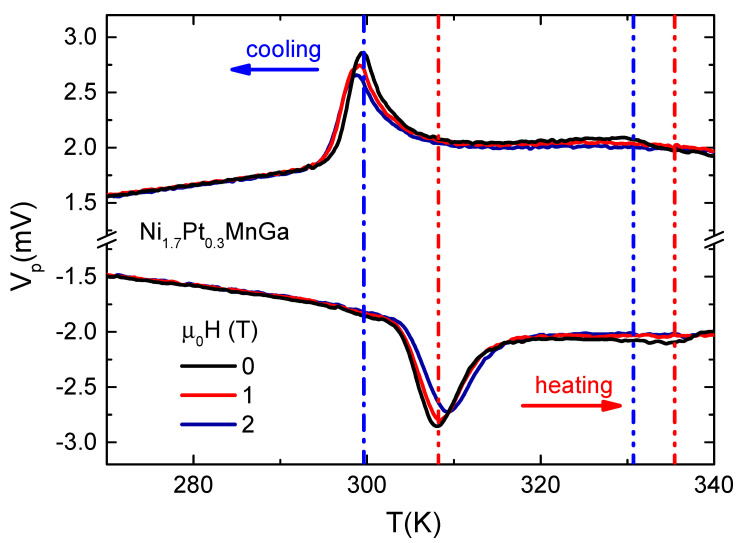
The Peltier voltage obtained with a rate of $T_{rate}=0.5$ K/min of Ni${}_{1.7}$Pt${}_{0.3}$MnGa at several fields. For H=0, the sharp peak is associated with a first order martensitin transition and the ferromagnetic–paramagnetic transition present a shoulder-like peak, which is located slightly above. The vertical dashed-dotted and dashed-dotted-dotted lines indicate the martensitic and ferromagnetic–paramagnetic transitions, respectively. The blue and red colors correspond to cooling and heating cycles, respectively.

The Peltier voltage, a value that is directly proportional to the heat capacity $C_{p}\left(T\right)=V_{p}/\left[T_{rate}A\left(T\right)\right]$[30], as a function of temperature for both cooling and heating processes for magnetic fields of 0, 1 and 2 T is depicted in Figure 4. Here $T_{rate}$ is the temperature sweeping rate, in our case 0.5 K/min. As expected, the Peltier voltage shows a peak around the martensitic transition, which is a signature of the presence of latent heat and the first-order nature of the transition. There is also a large hysteresis between the peak position on cooling and heating cycles. On heating (cooling), the application of a magnetic field shifts the transition to higher (lower) temperatures, with a shift of about 2 K for a magnetic field $\mu_{0}H=2$ T compared to zero field. For the paramagnetic–ferromagnetic transition the Peltier voltage does not show a peak, as expected for a second-order phase transition. Only a small shoulder is observed at 0 T, which is almost completely suppressed by the application of a magnetic field of 2 T. Moreover, a small thermal hysteresis of approximately 5 K is observed between the cooling and heating cycles for the paramagnetic–ferromagnetic transition.

## 4. Discussion

**Figure 5:**
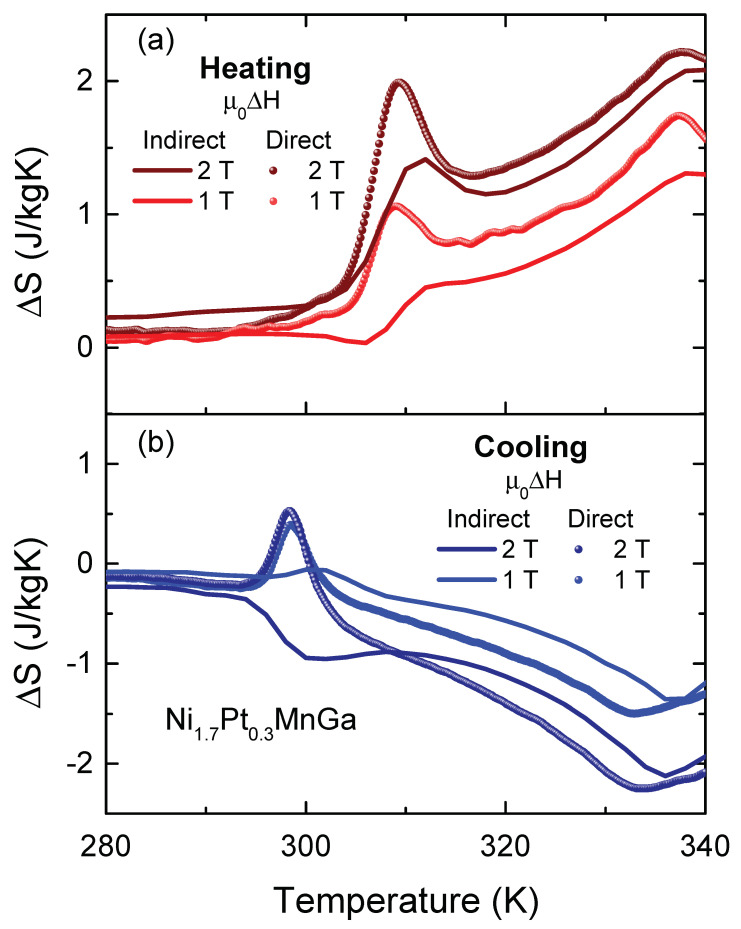
Temperature dependence of the isothermal entropy change, ΔS, in Ni${}_{1.7}$Pt${}_{0.3}$MnGa (**a**) upon heating and (**b**) upon cooling, determined directly from the heat flow and indirectly from magnetization.

We can calculate the heat capacity as well as the entropy change $\Delta S=\int_{0}^{T}\frac{C_{p}\left(T^{\prime }\right)}{T^{\prime }}dT^{\prime }$ from the $V_{p}$ curves [30], which allows us to directly compare the result of the heat-flow measurements with those from the magnetization data presented in Figure 3 and, therefore, to estimate the role played by irreversibilities and hysteresis effects on the properties of the MCE. The isothermal entropy change derived directly from the heat flow and that from the magnetization data are compared in Figure 5 for magnetic field variations $\mu_{0}\Delta H=1$ T and 2 T.

The behavior of ΔS measured in the temperature interval of the martensitic transformation upon heating and cooling is of particular interest because it can give insights into the importance of irreversible effects that are relevant for cooling applications. We first discuss the ΔS curves obtained directly from the heat-flow experiment (see Figure 5). In principle, one expects that ΔS recorded on cooling mimics the dependence of the isothermal magnetic entropy change on heating with just a change in the temperature position of the peaks, indicating the transitions. For the ferromagnetic–paramagnetic transition, the behavior for both cooling and heating processes is similar. In the heating processes, we find that $\Delta S_{FM}$ reaches 1.7J/kgK and 2.2J/kgK for magnetic field changes of 1 and 2 T respectively, at 338 K and in the cooling process the maximum of $\Delta S_{FM}$ appears at 333 K, with maximum values of 1.5J/kgK and 2.2J/kgK for magnetic field changes of 1 and 2 T, respectively. More interestingly, for the martensitic transition upon cooling, contrary to ΔS obtained from magnetization curves, we observe an inverse magnetocaloric effect at 299 K with $\Delta S_{M}$ of 0.4 J/kgK for $\mu_{0}\Delta H=1$ T and 0.5 J/kgK, for $\mu_{0}\Delta H=2$ T. The origin of the apparent discrepancy between the $\Delta S_{M}$ from cooling and heating cycles can be explained based on the strong hysteresis of the temperature and magnetic-field induced thermoelastic martensitic transformation in Ni${}_{1.7}$Pt${}_{0.3}$MnGa (see inset of Figure 2). Upon heating, around the martensitic transition temperature, the application of a magnetic field easily converts the martensite into ferromagnetic austenite, corresponding to the observed positive peak. Upon cooling, at zero magnetic field, there is a temperature-induced conversion of the sample to the martensite phase. In the applied magnetic field, it is necessary to determine the entropy change, and the converted sample does not return to the austenitic phase and the ‘negative’ MCE is governed by the magnetic properties of both phases.

We now compare the entropy change, determined based on magnetization curves and by the heat-flow method (see Figure 5). It is worth mentioning that both measurements were performed using the same measurement protocol, as described in Section 2. We observe that the peak positions in the entropy change from the heat-flow experiment agree reasonably well with those determined from the magnetization curves for the heating process. However, for the cooling cycle, the thermal hysteresis for the ferromagnetic–paramagnetic transition is larger in the heat-flow experiments. The fact that ΔS associated with the ferromagnetic–paramagnetic transition, obtained by the heat-flow method, is larger than that obtained from magnetization, can be attributed to the small thermal hysteresis observed at this transition, which is not detected by the indirect magnetization method. Moreover, we observed a significant difference in ΔS(T) around the martensitic transition, which is first-order in nature. We note an inverse MCE in the heat-flow data for both magnetic field changes, $\mu_{0}\Delta H=1$ T and 2 T. In ΔS(T) determined indirectly from magnetization data this is only observed for a magnetic field change of $\mu_{0}\Delta H=1$ T. In 2 T, we find a direct MCE. We argue that the difference in the ΔS(T) curves is associated with the first-order-type of the transition and the two different methods used to determine ΔS(T). It can be explained considering the paths of the experimental processes in the phase diagram of the compound. While the magnetization curves were obtained in temperature cycles with 0.2 T steps from 0 to 2 T, making the sample cross the phase boundaries, shown in the phase diagram in the inset of Figure 2, several times, the heat-flow measurements were recorded at constant fields of 1 and 2 T, respectively, i.e. each phase boundary was only crossed once.

## 5. Conclusions

In summary, we studied the magnetocaloric effect in the Heusler compound Ni${}_{1.7}$Pt${}_{0.3}$MnGa using a direct and an indirect method to determine the entropy change at the martensitic transformation. Our experimental results revealed that in the vicinity of the first-order martensitic phase transition the magnitude of the magnetic entropy change ΔS depends on the thermal history of the sample and the measuring method. Apparent discrepancies in ΔS(T) determined indirectly from magnetization curves and ΔS(T) obtained directly from heat-flow calorimeter experiments point directly at the weakness of the indirect method in the study of first-order type phase transitions. Our study exemplifies the power of heat-flow calorimeter technique based on Peltier elements for the investigation of first-order phase transitions.

## Data Availability

The data presented in this study are available on reasonable request from the corresponding author.

## References

[1] Felser C., Wollmann L., Chadov S., Fecher G.H., Parkin S.S.P. (2015). Basics and prospective of magnetic Heusler compounds. APL Mater..

[2] Singh S., D’Souza S.W., Nayak J., Caron L., Suard E., Chadov S., Felser C. (2016). Effect of platinum substitution on the structural and magnetic properties of *Ni*_2_*MnGa* ferromagnetic shape memory alloy. Phys. Rev. B.

[3] Wollmann L., Nayak A.K., Parkin S.S., Felser C. (2017). Heusler 4.0: Tunable Materials. Annu. Rev. Mater. Res..

[4] Nayak A.K., Nicklas M., Chadov S., Shekhar C., Skourski Y., Winterlik J., Felser C. (2013). Large Zero-Field Cooled Exchange-Bias in Bulk Mn_2_PtGa. Phys. Rev. Lett..

[5] Nayak A.K., Nicklas M., Chadov S., Khuntia P., Shekhar C., Kalache A., Baenitz M., Skourski Y., Guduru V.K., Puri A. (2015). Design of compensated ferrimagnetic Heusler alloys for giant tunable exchange bias. Nat. Mater..

[6] Meshcheriakova O., Chadov S., Nayak A.K., Roessler U.K., Kuebler J., Andre G., Tsirlin A.A., Kiss J., Hausdorf S., Kalache A. (2014). Large Noncollinearity and Spin Reorientation in the Novel Mn2RhSn Heusler Magnet. Phys. Rev. Lett..

[7] Singh S., D’Souza S.W., Nayak J., Suard E., Chapon L., Senyshyn A., Petricek V., Skourski Y., Nicklas M., Felser C. (2016). Room-temperature tetragonal non-collinear Heusler antiferromagnet Pt_2_MnGa. Nat. Commun..

[8] Reis R.D.d., Ghorbani Zavareh M., Ajeesh M.O., Kutelak L.O., Sukhanov A.S., Singh S., Noky J., Sun Y., Fischer J.E., Manna K. (2020). Pressure tuning of the anomalous Hall effect in the chiral antiferromagnet Mn_3_Ge. Phys. Rev. Mater..

[9] Manna K., Sun Y., Muechler L., Kübler J., Felser C. (2018). Heusler, Weyl and Berry. Nat. Rev. Mater..

[10] Singh S., Caron L., D’Souza S.W., Fichtner T., Porcari G., Fabbrici S., Shekhar C., Chadov S., Solzi M., Felser C. (2016). Large Magnetization and Reversible Magnetocaloric Effect at the Second-Order Magnetic Transition in Heusler Materials. Adv. Mater..

[11] Singh S., Dutta B., D’Souza S.W., Zavareh M.G., Devi P., Gibbs A.S., Hickel T., Chadov S., Felser C., Pandey D. (2017). Robust Bain distortion in the premartensite phase of a platinum-substituted Ni_2_MnGa magnetic shape memory alloy. Nat. Commun..

[12] Nayak A.K., Fischer J.E., Sun Y., Yan B., Karel J., Komarek A.C., Shekhar C., Kumar N., Schnelle W., Kübler J. (2016). Large anomalous Hall effect driven by a nonvanishing Berry curvature in the noncolinear antiferromagnet Mn_3_Ge. Sci. Adv..

[13] Kübler J., Felser C. (2014). Non-collinear antiferromagnets and the anomalous Hall effect. EPL.

[14] Sivaprakash P., Esakki Muthu S., Singh A.K., Dubey K., Kannan M., Muthukumaran S., Guha S., Kar M., Singh S., Arumugam S. (2020). Effect of chemical and external hydrostatic pressure on magnetic and magnetocaloric properties of Pt doped Ni_2_MnGa shape memory Heusler alloys. J. Magn. Magn. Mater..

[15] Kulkova S.E., Eremeev S.V., Kakeshita T., Kulkov S.S., Rudenski G.E. (2006). The Electronic Structure and Magnetic Properties of Full- and Half-Heusler Alloys. Mater. Trans..

[16] Waske A., Dutta B., Teichert N., Weise B., Shayanfar N., Becker A., Hütten A., Hickel T. (2018). Coupling Phenomena in Magnetocaloric Materials. Energy Technol..

[17] Marcos J., Mañosa L., Planes A., Casanova F., Batlle X., Labarta A. (2003). Multiscale origin of the magnetocaloric effect in Ni-Mn-Ga shape-memory alloys. Phys. Rev. B.

[18] Liu J., Gottschall T., Skokov K.P., Moore J.D., Gutfleisch O. (2012). Giant magnetocaloric effect driven by structural transitions. Nat. Mater..

[19] Ghahremani M., Aslani A., Hosseinnia M., Bennett L.H., Della Torre E. (2018). Direct and indirect measurement of the magnetocaloric effect in bulk and nanostructured Ni-Mn-In Heusler alloy. AIP Adv..

[20] Palacios E., Bartolomé J., Wang G., Burriel R., Skokov K., Taskaev S., Khovaylo V. (2015). Analysis of the Magnetocaloric Effect in Heusler Alloys: Study of Ni_50_CoMn_36_Sn_13_ by Calorimetric Techniques. Entropy.

[21] Devi P., Ghorbani Zavareh M., Mejía C.S., Hofmann K., Albert B., Felser C., Nicklas M., Singh S. (2018). Reversible adiabatic temperature change in the shape memory Heusler alloy *Ni*_2.2_*Mn*_0.8_*Ga*: An effect of structural compatibility. Phys. Rev. Mater..

[22] Chabri T., Ghosh K., Mukherjee D., Nath T.K. (2020). Role of interplay of austenite and martensite phase fractions on the magnetocaloric and magnetoresistance effects across the martensite transition in Ni_45_Mn_44_Sn_7_In_4_ Heusler alloy near room temperature. J. Appl. Phys..

[23] Cicek M., Saritas S., Yildirim O., Emre B. (2020). Effect of the low constituent boron on martensitic transformation, magnetic, and magnetocaloric properties of Ni_50_Mn_35_In_15_ Heusler alloys. J. Alloy. Compd..

[24] Basso V., LoBue M., Sasso C.P., Bertotti G. (2006). Thermodynamic aspects of magnetic-field-driven phase transformations in Gd-Si-Ge alloys. J. Appl. Phys..

[25] Basso V., Küpferling M., Sasso C.P., Giudici L. (2008). A Peltier cell calorimeter for the direct measurement of the isothermal entropy change in magnetic materials. Rev. Sci. Instrum..

[26] Zavareh M.G., Mejia Salazar C., Nayak A.K., Skourski Y., Wosnitza J., Felser C., Nicklas M. (2015). Direct measurements of the magnetocaloric effect in pulsed magnetic fields: The example of the Heusler alloy Ni_50_Mn_35_In_15_. Appl. Phys. Lett..

[27] Salazar Mejia C., Zavareh M.G., Nayak A.K., Skourski Y., Wosnitza J., Felser C., Nicklas M. (2015). Pulsed high-magnetic-field experiments: New insights into the magnetocaloric effect in Ni-Mn-In Heusler alloys. J. Appl. Phys..

[28] Pfeuffer L., Gottschall T., Faske T., Taubel A., Scheibel F., Karpenkov A.Y., Ener S., Skokov K.P., Gutfleisch O. (2020). Influence of the martensitic transformation kinetics on the magnetocaloric effect in Ni-Mn-In. Phys. Rev. Mater..

[29] Plackowski T., Wang Y., Junod A. (2002). Specific heat and magnetocaloric effect measurements using commercial heat-flow sensors. Rev. Sci. Instr..

[30] Monteiro J.C.B., dos Reis R.D., Mansanares A.M., Gandra F.G. (2014). Determination of the magnetocaloric entropy change by field sweep using a heat flux setup. Appl. Phys. Lett..

[31] Monteiro J., Lombardi G., dos Reis R., Freitas H., Cardoso L., Mansanares A., Gandra F. (2016). Heat flux measurements of Tb_3_M series (M=Co, Rh and Ru): Specific heat and magnetocaloric properties. Phys. B Condens. Matter..

[32] De Sousa V., Monteiro J., dos Reis R., Medina A., Gama S., von Ranke P., Gandra F. (2012). Heat flow measurements and the order of the magnetic transition in (Dy,Gd)Co_2_ solid solutions. J. Alloy. Compd..

[33] Monteiro J.B., dos Reis R., Gandra F.G., Dilley N. Determination of the Magnetocaloric Properties Using a PPMS. Quantum Design Application Note 1085-200. https://www.qdusa.com/siteDocs/appNotes/1085-200.pdf.

